# A Novel *De Novo* Heterozygous Missense Variant in *CTNNB1* (c.2215A>C, p.Met739Leu) Underlying Neurodevelopmental Disorder With Spastic Diplegia and Visual Defects (NEDSDV): A Case Report

**DOI:** 10.1002/ccr3.73743

**Published:** 2026-10-16

**Authors:** Omar Jamal Ibrahim, Ali Rajabi, Mortaza Bonyadi, Mohammad Barzegar

**Affiliations:** ^1^ Department of Animal Biology, Faculty of Natural Sciences University of Tabriz Tabriz Iran; ^2^ Pediatric Health Research Center, Mardani Azari Children Hospital Tabriz University of Medical Sciences Tabriz Iran; ^3^ Center of Excellence for Biodiversity, Faculty of Natural Sciences University of Tabriz Tabriz Iran

**Keywords:** *CTNNB1*: c.2215A>C, *de novo* mutation, neurodevelopmental disorder with spastic diplegia and visual defects (NEDSDV), p.Met739Leu, whole exome sequencing

## Abstract

We report a 7‐year‐old male with microcephaly, spasticity, strabismus, dysmorphism, autistic behaviors, and moderate ID due to a novel *de novo* likely pathogenic *CTNNB1* variant (c.2215A>C, p.Met739Leu). This case underscores Whole Exome Sequencing's diagnostic utility for early detection of rare monogenic neurodevelopmental disorders.

## Key Clinical Message

A novel *de novo* heterozygous *CTNNB1* likely pathogenic missense variant, c.2215A>C (p.Met739Leu), expands the genetic spectrum of NEDSDV. Consider *CTNNB1* testing, preferably whole‐exome sequencing, in children with developmental delay, spastic diplegia, visual defects, microcephaly, and autistic behavior to enable early diagnosis and multidisciplinary care.

## 1 Introduction

Neurodevelopmental disorder with spastic diplegia and visual defects (NEDSDV; OMIM: #615075) is a rare monogenic neurodevelopmental disorder NDD caused by a *CTNNB1* gene heterozygous mutation. It is characterized by developmental delay, intellectual disability, visual defects, axial hypotonia, and progressive muscle stiffness, especially in the legs. Behavioral problems including autism spectrum disorder, inattention, hyperactivity, aggression, self‐mutilation, and sleep disturbances are also recognized as common features. There are currently no established treatments, and management includes mainly supportive care [1, 2].

Catenin beta 1 (*CTNNB1*, NM_001904) is an important gene that produces beta‐catenin protein and is located on chromosome 3p22. Its typical transcript contains 14 protein‐coding exons, which encode β‐catenin consisting of 781 amino acids [3]. *CTNNB1* was initially described as a potential tumor suppressor gene and is linked to tumor development in a variety of cancers [4]. Further studies also suggest its role in the CNS, especially in cortical development, neural stem cell proliferation and neurogenesis, neuronal apoptosis, cell adhesion, and synaptic connection [5]. This protein is a key factor in the development of the nervous system, mainly for two reasons: it is central to the Wnt/β‐catenin pathway, and it is involved in the morphogenesis of epithelial and nervous tissue, particularly as part of adherens junctions between cells. Cell–cell junctions have a decisive influence on the mechanical properties of tissues and are also essential for neuronal synapses and synaptic plasticity, which underlie memory and learning [6]. Several studies have demonstrated the role of this protein in the emotional learning process and declarative memory [7]. Mutations of *CTNNB1* lead to the dysfunction of the Wnt signaling pathway that regulates gene transcription and further disturbs synaptic plasticity, neuronal apoptosis, and neurogenesis [5]. Patients with CTNNB1‐related neurodevelopmental disorder show *de novo* CTNNB1 variants with a broad spectrum of clinical features, such as developmental delay and intellectual disability [8].

Here, we present a clinically and molecularly confirmed case of NEDSDV due to a novel heterozygous missense variant of *CTNNB1*:C.2215A>C (p.Met739Leu), identified via WES and validated by Sanger sequencing.

## 2 Case Report

The patient is a 7‐year‐old male who was born at 36 weeks' gestation following an uncomplicated pregnancy via normal vaginal delivery, without perinatal problems. At birth, the head circumference was 32 cm, the birth body weight was 3200 g, and the birth length was 47 cm. He is the first child of non‐consanguineous healthy parents (the mother 26 years and father 30 years), without a family history of neurologic or developmental disorder. After the neonatal period, the parents noticed truncal hypotonia. He achieved developmental milestones with delay: independent walking at age 18 months and a few meaningful words at age 5 years. Physical and neurological examination at age 7 years revealed: microcephaly, head circumference 48 cm (lower than the 3rd percentile), body weight 20 kg (‐2SD), length 120 cm (‐2SD), truncal hypotonia with peripheral spasticity, hyperactive deep tendon reflexes, and toe‐toe walking. Dysmorphic features such as epicanthal fold, high arched palate, thin upper lip, long and smooth philtrum, abnormal hairline, and small alae nasi were seen (Figure 1). In neurodevelopmental evaluation, he had: cognitive impairment as moderate intellectual disability, without complete self‐care; he was dependent on his mother for feeding, without toilet training, with a significant speech delay; he expressed only a few meaningful words, without the ability to make a sentence, and behavioral problems such as autistic behavior, restlessness, and sleep disturbances. On ophthalmologic examination, strabismus and hypermetropia were observed; he wears glasses. Neurometabolic studies included thyroid function tests, creatine phosphokinase, liver function tests, cholesterol, and uric acid, which are within normal limits. Electrodiagnostic study was normal. Brain MRI was performed at age 5 years and was reported as normal. On ophthalmologic examination, strabismus and hypermetropia were observed, so he wears glasses.

**FIGURE 1:**
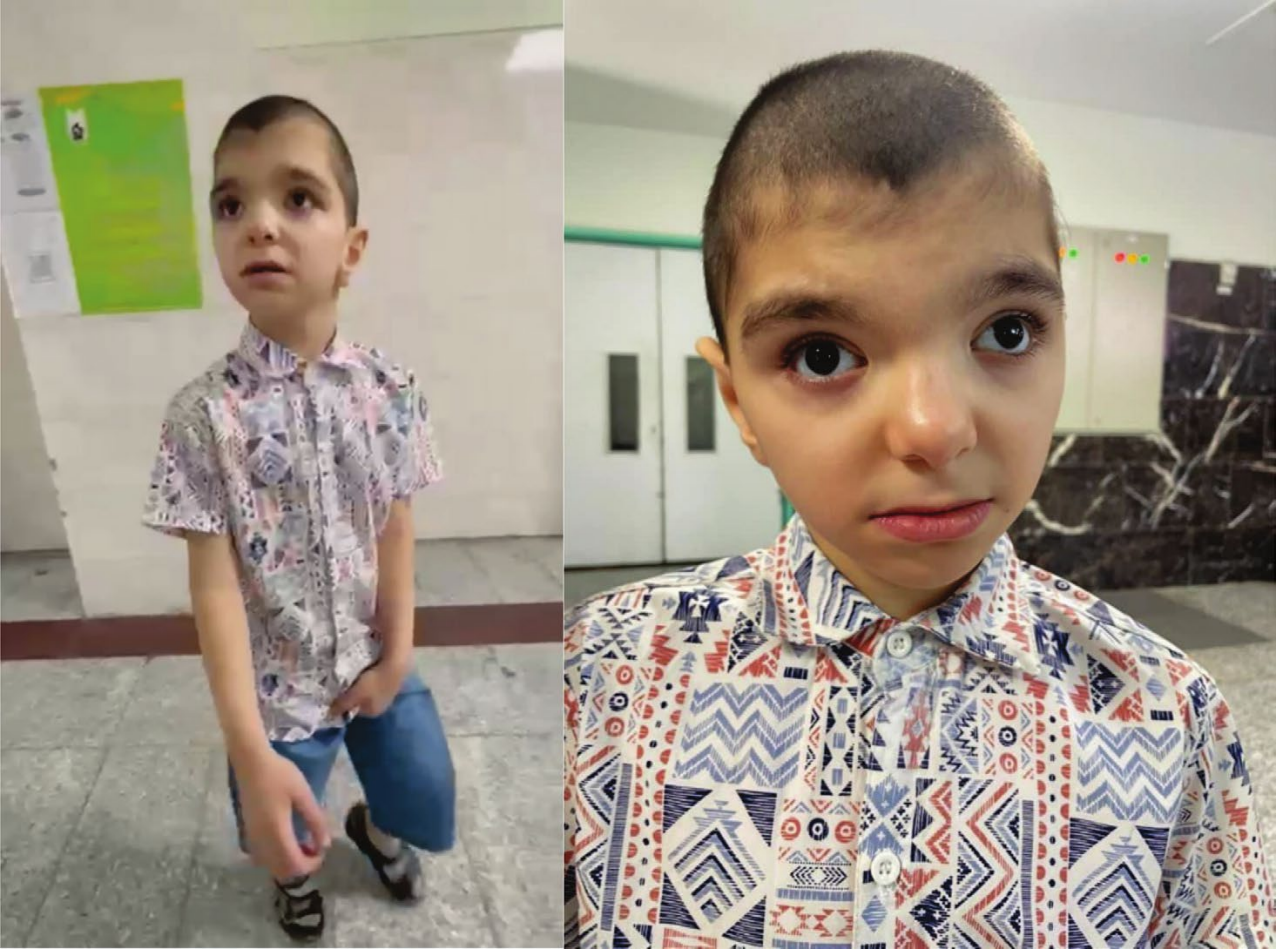
Photograph of patient at age 7 years. Left: Full body view. Right: Magnified view of the head and face. Dysmorphic features: Bulbous nasal tip, thin upper lip, small alae nasi, long and smooth philtrum, epicanthal folds, and abnormal hairline (forehead hirsutism).

Based on these findings, the differential diagnosis was hereditary spastic paraplegia (complicated) and leukodystrophy. For confirmation of diagnosis, genetic studies were done. Finally, based on the results of WES, the diagnosis of Neurodevelopmental Disorder with Spastic Diplegia and Visual defect (NEDSDV) was made. Currently, the patient is under a multidisciplinary rehabilitation program, which includes physical therapy, occupational therapy, and speech therapy as part of an outpatient program. We have followed the patient regularly in our multidisciplinary clinic during the last 6 months.

## 3 Genetic Studies

The male, 7‐year‐old child was referred to the Mardani Azari Children's Hospital, Tabriz, Iran. This study was conducted in accordance with the Declaration of Helsinki and approved by the Ethics Committee of Tabriz University of Medical Sciences (approval code: IR.TBZMED.FMD.REC.1404.397). Written informed consent was obtained from the patient's parents for participation in this study and for publication of this case report.

### 3.1 Whole‐Exome Sequencing

Blood samples of the patient and his parents were collected in EDTA tubes. The genomic DNA was isolated using the standard salting‐out method, quantified, and subjected to WES analysis using the Agilent SureSelect Human All Exon V7 Target Enrichment kit according to the manufacturer's protocol. The enriched library was sequenced on an Illumina NovaSeq 6000 platform, generating 150‐bp paired‐end reads. The results showed a pathogenic, heterozygous, missense variant in the *CTNNB1* gene (C.2215A>C p.Met739Leu) as the most plausible candidate. Parental testing did not harbor the variant.

The Mean Depth of Coverage (Mean Depth of Coverage) was considered to be at least 30× to ensure that most exome regions are read with sufficient accuracy. For regions with low coverage and for validation, supplementary sequencing was performed using the Sanger method using the ABI 3500 Genetic Analyzer (Applied Biosystems). Reads were aligned to the human reference genome GRCh37/hg19 using Burrows‐Wheeler Aligner (BWA) to generate SAM files. They were then converted to sorted BAM files using SAMtools (Figure 2a). Raw data quality assessment was performed using FastQC software.

**FIGURE 2:**
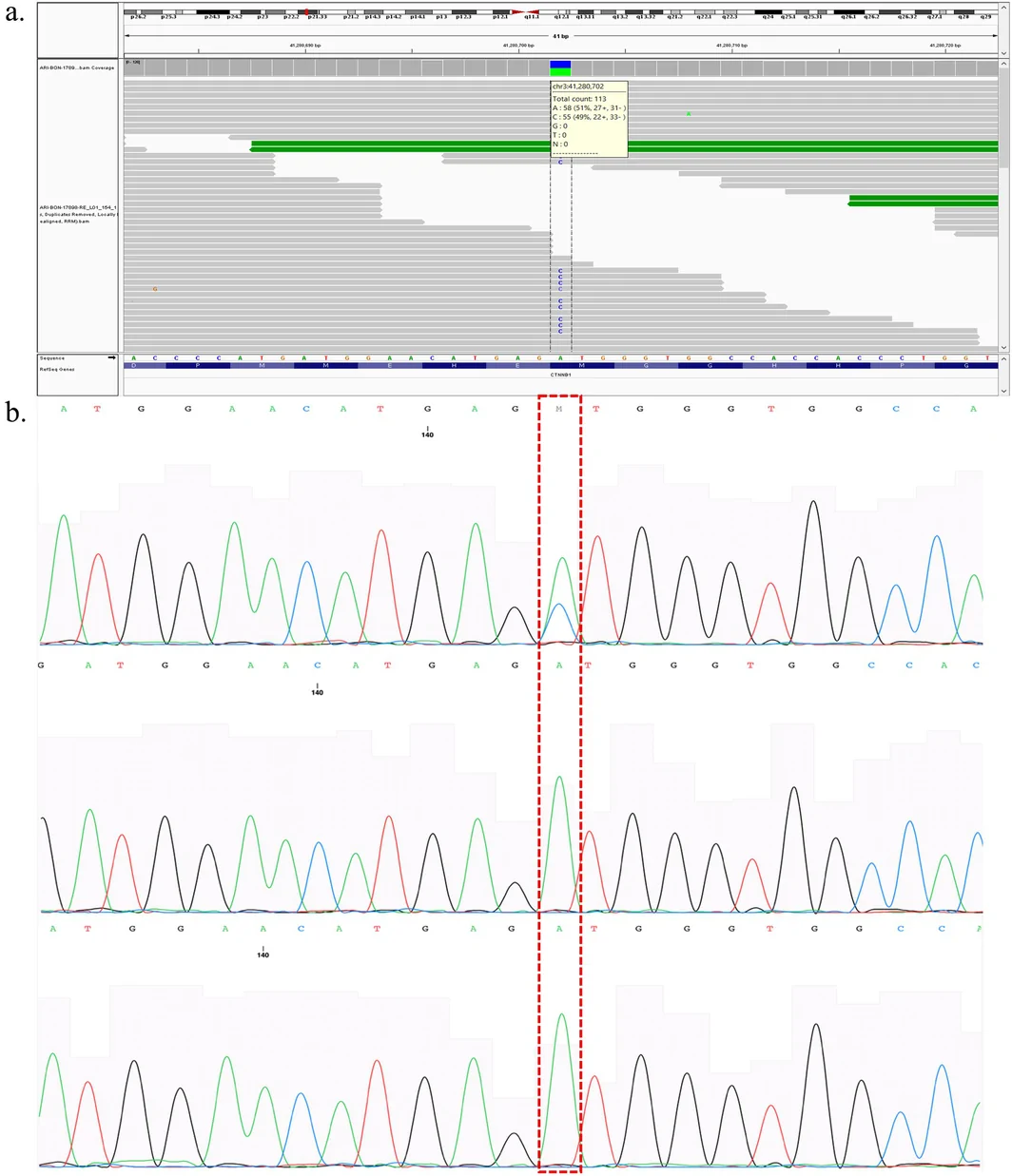
Molecular characterization of the CTNNB1 c.2215A>C (p.Met739Leu) variant. (a) Integrative Genomics Viewer (IGV) screenshot of (WES) data from the proband's BAM file, demonstrating the heterozygous A>C substitution at nucleotide position c.2215 (NM_001904.4) in the CTNNB1 gene with balanced allelic reads (A: 58 [51%], C: 55 [49%]) and high base call quality (Q > 40). (b) Sanger sequencing chromatograms confirm the heterozygous variant in the proband (arrow). The de novo mutation was confirmed through conventional Sanger sequencing in the patient (Upper graph), the father (Middle graph), and the mother (Lower graph). Both parents are wild‐type homozygous, confirming de novo inheritance.

To determine the pathogenicity of this novel variant, the American College of Medical Genetics and Genomics (ACMG) guideline was utilized [9]. The *CTNNB1* c.2215A>C variant (NM_001904.4; p.Met739Leu) was classified as Likely Pathogenic according to the ACMG/AMP guidelines, supported by PM2 (extremely low population frequency, with an allele frequency of 0.0% in gnomAD and no homozygous individuals observed, remaining well below the gene‐specific BA1 and BS1 thresholds of 0.5% and 0.1%, respectively), PP4 (the patient's phenotype of severe intellectual disability, progressive microcephaly, ataxia, spasticity, and mild facial anomalies is highly specific for *CTNNB1*‐related severe intellectual disability‐progressive spastic diplegia syndrome), PM6 (*de novo* in a patient with phenotype consistency, no family history and both maternity and paternity are assumed.), and PP2 (the variant is a missense substitution in *CTNNB1*, a gene with a low rate of benign missense variation and for which missense mutations are a known common disease mechanism), whereas PM1, BA1, BS1, and BS2 were not met.

### 3.2 Segregation Analysis and Sanger Sequencing

The presence of the c.2215A>C variant was validated by Sanger sequencing using the following primers: Forward: 5′‐TGGATGCCCTAACCTCAGTGT‐3′ (17898‐CTNNB1‐F); Reverse: 5′‐CAGATCTGGCAGCCCATCAA‐3′ (17898‐CTNNB1‐R).

PCR was performed at an annealing temperature of 60°C, producing an amplicon of 229 bp. PCR repeats are marked and removed using Picard. The sequencing reaction was performed using the forward primer, and the chromatogram confirmed the heterozygous A>C substitution at position c.2215 (Het, AD, p.Met739Leu NM_001904.4, Figure 2b). The variant was localized to the armadillo repeat domain of the β‐catenin protein. The identified variant is characterized as shown in the Table 1.

**TABLE 1:** Genetic characteristics of the CTNNB1 c.2215A>C (p.Met739Leu) variant.

Parameter	Detail
Gene	CTNNB1
Transcript	NM_001904.4
cDNA position	2429
CDS position	2215
Nucleotide change	c.2215A>C
Codon change	ATG>CTG
Amino acid change	p.Met739Leu (Methionine → Leucine)
Variant type	Missense
Zygosity	Heterozygous
Inheritance	Autosomal dominant (AD)
CDS end	The variant is located near the C‐terminal region of the coding sequence.

## 4 Discussion

NEDSDV is typically caused by *de novo*, heterozygous, and loss‐of‐function variants of CTNNB1 [8]. This autosomal dominant disorder is characterized by syndromic intellectual disability, global developmental and motor delay, very limited speaking ability, microcephaly, spasticity (particularly in lower limbs), visual abnormalities, and in some cases, behavioral abnormalities [10]. Our case reports a 7‐year‐old boy presenting with microcephaly, truncal hypotonia with peripheral spasticity, mild to moderate intellectual disability, significant speech delay, strabismus and hypermetropia, and behavioral problems. These clinical characteristics are highly consistent with the phenotypic spectrum of NEDSDV. In addition, normal brain MRI and metabolic investigations further support a genetic neurodevelopmental etiology of the disorder.

This case provides a detailed clinical and molecular characterization of a patient with NEDSDV reporting a novel missense variant in the *CTNNB1* gene. This gene is 67,646 nucleotides long with 15 exons [11]. This sequence change replaces methionine, which is neutral and non‐polar, with leucine, which is neutral and non‐polar, at codon 739 of the CTNNB1 protein (p.Met739Leu). To date, the ClinVar database includes 106 genetic variations associated with NEDSDV. The majority of these variations are loss‐of‐function, whereas missense variations are less commonly reported [12]. This variant was not previously reported in ClinVar or other publicly available disease databases; however, it is classified as likely pathogenic according to ACMG/AMP guidelines. Further functional studies are required to determine its precise biological consequences.

*De novo* mutations are enriched in genes involved in the Wnt signaling pathway, including *CTNNB1*, which regulates cell growth and cell adhesion. By interacting with N‐cadherin, CTNNB1 shapes synaptic structure and regulates excitatory postsynaptic strength. In addition, the axonal localization and translation of CTNNB1 modulate presynaptic vesicle release. Loss‐of‐function mutations of *CTNNB1* were linked to intellectual disability [13].

WES has been used as a method of gene discovery in large series of patients with NDDs. Mutations in hundreds of known genes give rise to rare variants, and *de novo* mutations are particularly enriched in genes participating in critical neurodevelopmental pathways [14, 15]. In this report, the WES method was used to identify this novel *CTNNB1* variation, which was subsequently validated by Sanger sequencing.

The first cases of NEDSDV were described in 2012 by de Ligt et al. using exome sequencing to identify germline, *de novo*, and likely pathogenic variants in *CTNNB1* [16]. In later years, features such as retinopathy and vitreoretinopathy were considered as symptoms of this disorder [17]. In a recent study by Kayumi et al. [18], the genomic and phenotypic characterization of 404 patients with neurodevelopmental disorders caused by CTNNB1 variants was provided. The results were mainly obtained through exome sequencing. The findings of this investigation confirm that NEDSDV is a clinically homogeneous disorder with consistent neurological traits [18]. Ho et al. showed the CTNNB1‐related NEDSDV in the Chinese population [19], Dashti et al. reported a novel *de novo* nonsense mutation in the CTNNB1 gene in an Iranian girl [20], and Lee et al. identified NEDSDV cases in Korean patients with an extended clinical and genetic spectrum [8]; these results demonstrate the global distribution of this condition. Another missense and single amino acid substitution variant in CTNNB1 (c.1723G>A, p.G575R) was introduced by Rossetti et al. [17], which demonstrated significant vitreoretinopathy [17].

In this report, we confirmed a case of a 7‐year‐old child with NEDSDV caused by a novel heterozygous missense variant, *CTNNB1* c.2215A>C p.Met739Leu, identified by WES and validated by Sanger sequencing. This case expands the allelic heterogeneity of *CTNNB1*‐related disorders and demonstrates the power of a sequential WES‐to‐Sanger diagnostic pipeline.

## 5 Conclusion

In children with global developmental delay or intellectual disability, especially with additional manifestations including truncal hypotonia, peripheral spasticity, behavioral problems, post‐natal microcephaly, and ophthalmic abnormalities, although the perinatal period is normal and neuroimaging and metabolic workup show nonspecific findings, *CTNNB1*‐associated intellectual disability can be considered as a differential diagnosis.

## Author Contributions

**Omar Jamal Ibrahim:** investigation, writing – original draft. **Ali Rajabi:** methodology, validation, writing – review and editing, writing – original draft. **Mortaza Bonyadi:** conceptualization, writing – original draft, writing – review and editing, validation, supervision. **Mohammad Barzegar:** conceptualization, writing – original draft, writing – review and editing, supervision.

## Funding

The authors have nothing to report.

## Ethics Statement

This study was conducted in accordance with the Declaration of Helsinki and approved by the Ethics Committee of Tabriz University of Medical Sciences (approval code: IR.TBZMED.FMD.REC.1404.397).

## Consent

Written informed consent was obtained from the patient's parents for participation in this study and for publication of this case report.

## Conflicts of Interest

The authors declare no conflicts of interest.

## Data Availability

The data that support the findings of this study are available from the corresponding author upon reasonable request.
